# Relationship between nasal septum morphology and nasal obstruction symptom severity: computed tomography study

**DOI:** 10.1016/j.bjorl.2020.09.004

**Published:** 2020-10-10

**Authors:** Natasa Janovic, Aleksa Janovic, Biljana Milicic, Marija Djuric

**Affiliations:** a University of Belgrade, Faculty of Medicine, Institute of Anatomy, Belgrade, Serbia; b University of Belgrade, Faculty of Dental Medicine, Department of Diagnostic Radiology, Belgrade, Serbia; c University of Belgrade, Faculty of Dental Medicine, Department of Statistics, Belgrade, Serbia

**Keywords:** Nasal septal deviation, Morphology, Computed tomography, Nasal obstruction, NOSE scale

## Abstract

**Introduction:**

The impact of the nasal septum morphology on the severity of obstruction symptoms has not been fully explored.

**Objective:**

This study aimed to investigate whether the morphology of the deviated nasal septum assessed by computed tomography may explain nasal obstruction severity.

**Methods:**

The study included 386 patients who were referred to the computed tomography examination of the paranasal sinuses. Patient selection criteria were the absence of facial anomalies, facial trauma, nasal surgery, and sinonasal tumors. Computed tomography images were used to estimate deviated nasal septum prevalence, the prevalence of Mladina's seven types of deviated nasal septum, and to measure the deviated nasal septum angle. Nasal obstruction severity was assessed by the nasal obstruction symptom evaluation, NOSE scale. The relationship between NOSE score, deviated nasal septum morphology, and deviated nasal septum angle was performed by a statistical regression model on the reduced sample of 225 patients.

**Results:**

The prevalence of deviated nasal septum was 92.7%. Type 7 deviated nasal septum was the most frequent (34.2%) followed by type 5 (26.2%) and type 3 (23.6%). The worst NOSE scores were recorded in the type 2 deviated nasal septum (45.00 ± 28.28). The mean deviated nasal septum angle in patients with nasal obstruction was 8.5° ± 3.24. NOSE scores were not significantly associated with deviated nasal septum types and angles.

**Conclusion:**

Patients with different types of deviated nasal septum have different NOSE scores. Computed tomography morphology of the deviated nasal septum could not fully explain the severity of nasal obstruction.

## Introduction

Nasal septal deviation (NSD) is a frequent finding in both clinical and radiological examinations, with prevalence up to 89.2% in the general population.^1^ Patients with NSD-related nasal obstruction are often candidates for septoplasty.^2^ Surgical correction of the deviated nasal septum is the most frequent procedure performed in adults by otorhinolaryngologists.^2^, ^3^, ^4^ Recent epidemiological studies reported that 260,000 and 10,000−95,000 septoplasties are performed annually in the USA and European countries respectively.^2^, ^5^, ^6^, ^7^

Clinicians and, more recently, health insurance companies have been searching for a diagnostic method that could serve as an objective indicator of the nasal obstruction severity, and thus strongly supporting the decision for septoplasty. Computed tomography (CT) was suggested as a method of choice to give detailed insight into the septum morphology and to provide one or more parameters that could potentially predict nasal obstruction severity. However, the few studies that correlated CT images and subjective nasal obstruction symptoms reported conflicting results about which morphological characteristics of the NSD, if any, should be recommended as an indicator of the symptom severity.^8^, ^9^, ^10^ Besides contradictory results, CT was used only for NSD angle and cross-sectional area measurement in the different parts of the nasal cavity. An advantage of the CT to show the detailed morphology of the deviated septum was not fully utilized. It is still unclear whether the complex morphology of NSD (e.g. spurs, S-shaped, “gutter” form) may have any influence on the subjective sensation of the nasal obstruction severity.

Although there are numerous classifications of NSD in clinical practice, Mladina's classification is considered the most detailed.^1^ This comprehensive classification includes complicated anatomical variants of the nasal septum usually omitted by other simplified classifications and divides NSD into seven types.^1^

This study aimed to investigate the association between nasal obstruction symptom severity assessed by the NOSE scale and CT morphology of NSD and the angle of NSD. Anatomical variants of the NSD, including spurs, S-shaped, and “gutter” form of NSD were classified according to Mladina's classification. An additional goal of the study was to estimate the prevalence of NSD in the Serbian population. We hypothesized that the morphology of the NSD may explain the severity of nasal obstruction symptoms and that patients with a higher angle of NSD have more severe nasal obstruction.

## Methods

### Patients

This study was conducted at the Department of Diagnostic Radiology. A total of 386 participants were successively selected among patients referred to the CT examination of the maxillofacial region due to various rhinologic or odontogenic diseases. The sample size was calculated according to the standard method for prevalence studies.^11^, ^12^ Patient selection criteria for this part of the study were: the nasal cavity in the field of view on CT scans, the absence of facial anomalies, history of facial trauma, nasal surgery, and sinonasal tumors. All participants were older than 18 years and gave written informed consent for participation in the study. The study was approved by the Ethics Committee of the Faculty of Medicine, nº 29/V-1.

### CT examination

CT examination was performed on Siemens Somatom Sensation 16 device (Munich, Germany). Patients were lying in a supine position. The Frankfurt’s line of the head was set perpendicular to the horizontal plane. Patients were scanned in 3 mm thick axial sections (tube current 270 mAs, voltage of 120 kV). Additional multi-planar reformation images were reconstructed from the raw data in 0.75 mm thick axial sections using bone window settings.

### CT evaluation of NSD

The presence of the NSD on CT images was recorded and classified by an experienced head and neck radiologist. Classification of the NSD was made according to Mladina’s classification scheme.^1^ Briefly, types 1 and 2 are deviations of the anterior or cartilaginous part of the nasal septum in the nasal valve region. Type 3 is situated inside the nasal cavity at the level of the middle turbinate that resembles the letter C. Type 4 is double curved septum similar to the letter S. The anterior curve is usually placed in the region of the nasal valve, while the posterior curve is situated more inside in the nasal cavity. Type 5 is located in the bony septum and contains a bony spur. Type 6 is parallel to the horizontal plate that has a protuberance on one side and gutter on the other side. Type 7 is a combination of the two or more abovementioned types.

**Figure 1:**
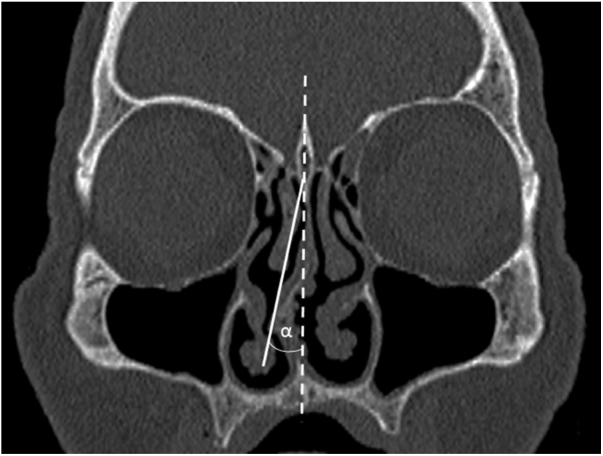
Measurement of the NSD angle on coronal CT image.

The maximum degree of the NSD angle was also measured directly on the Siemens CT workstation. The angle was measured on the single coronal CT section where NSD was the most prominent. The standard CT technique for the NSD angle measurement was applied (Fig. 1).^8^, ^13^ Briefly, the angle was measured between two lines of reference. The first line running from the nasal septum insertion in the maxillary crest and the junction point of the perpendicular and the cribriform plate corresponded to the midline. The second line was run from the junction point of the perpendicular and the cribriform plate to the most prominent point of the NSD. For double curved NSD (type 4) greater value was taken into account.

### Nasal obstruction symptom evaluation

In order to investigate the association between nasal obstruction severity and septum morphology, additional exclusion criteria were applied. Patients who had a history and/or CT finding of any condition that may cause nasal obstruction were excluded from the first study group. These conditions included: anatomical variations of the nasal cavity structures (e.g. paradoxical middle turbinate, concha bullosa), turbinate hypertrophy, rhinosinusitis, nasal polyps, adenoid hypertrophy, asthma, chronic obstructive lung disease. Therefore, the study group was reduced to 225 patients. These patients fulfilled the NOSE questionnaire validated for the Serbian population (Table 1) and self-assessed severity of the nasal obstruction experienced in the last month.^14^ A sum of the five-question responses is calculated and multiplied by 5 so that the total NOSE score had a range between 0 (no obstruction) and 100 (the most severe obstruction).^15^

**Table 1:** The Serbian version of the Nasal Obstruction Symptom Evaluation (NOSE-s) scale.

Over the past 1 month, how much of a problem were the following conditions for you?						
*У последњих месец дана, колики проблем су Вам представљале следеће тегобе?*						
Please circle the most correct response:						
*Mолимо Вас да заокружите одговор који најбоље описује Ваше тегобе*						
		Not a problem	Very mild problem	Moderate problem	Fairly bad problem	Severe problem
		*Без тегоба*	*Веома благе тегобе*	*Cредње изражене тегобе*	*Изражене тегобе*	*Веома изражене тегобе*
1.	Nasal congestion or stuffiness	0	1	2	3	4
	*Oсећај запушености носа*					
2.	Nasal blockage or obstruction	0	1	2	3	4
	*Oсећај непроходности носа*					
3.	Trouble breathing through my nose	0	1	2	3	4
	*Oтежано дисање кроз нос*					
4.	Trouble sleeping	0	1	2	3	4
	*Лош сан*					
5.	Unable to get enough air through my nose during exercise or exertion	0	1	2	3	4
	*Oтежано дисање кроз нос приликом изражене физичке активности*					

### Statistical analysis

Statistical analyses were performed in SPSS for Windows, version 15.0 (SPSS, Inc., Chicago, IL). The Kolmogorov-Smirnov test assessed the normality of data distribution. The severity of the nasal obstruction symptoms expressed by NOSE score and parameters obtained from CT images of the nasal cavity were evaluated using appropriate descriptive statistical methods. Frequencies of septal deviation among genders were compared by Chi-square test. The Kruskal-Wallis test was used to analyze NOSE scores in relation to the different types of NSD. Association between NOSE scores, types of NSD, and angle of NSD were thoroughly analyzed using linear regression analysis. The power of the study was 0.8. The statistical significance was set at a level of 0.05.

## Results

### Estimation of NSD prevalence

Among 386 patients who participated in the first part of the study, 153 (39.6%) were males, and 233 (60.4%) were females. Mean age was 55.08 ± 16.09 years (mean ± SD). The estimated prevalence of NSD was 92.7% (358/386). The prevalence of each type of NSD according to Mladina's classification system in this population is presented in Table 2. The most prevalent type was type 7 (34.9%). Types 5 and 3 were also frequent, with percentages of 24.9% and 23.7%, respectively. The least frequent type was type 2 (0.6%) (Table 2). The Chi-square test showed no significant difference in NSD frequencies among genders (Pearson Chi-Square = 1.545, *p* = 0.214).

**Table 2:** Prevalence of NSD types according to Mladina's classification.

Type of NSD	Number of patients (%)
Type 1	11 (3.1%)
Type 2	2 (0.6%)
Type 3	85 (23.7%)
Type 4	40 (11.2%)
Type 5	89 (24.9%)
Type 6	6 (1.7%)
Type 7	125 (34.9%)
Total	358 (100%)

### Association between Mladina's NSD types and NOSE scores

Table 3 displays the frequencies of each Mladina's type of NSD in the group of 225 patients who participated in the second part of the study. The similar prevalence distribution of NSD types was noted as in the original study population. The most common was type 7, while the least frequent was type 2 (Table 3). The mean NOSE scores for all 225 patients as well as the mean NOSE scores in each type of NSD are also shown in Table 3. According to NOSE scores, patients with type 2 NSD reported the worst symptoms with the mean value of 45.00 ± 28.28 (Table 3). As confirmed by the Kolmogorov-Smirnov test, the distribution of the NOSE score data was not normal (*p* < 0.001). Observed differences in NOSE scores among different NSD types were not statistically significant (Chi-Square 7.303, *p* = 0.294).

**Table 3:** Distribution of NSDs types and NOSE scores for 225 patients according to Mladina's classification.

Type of NSD	Number of patients (%)	NOSE score	
		Mean ± SD	Median
Type 1	4 (1.8%)	20.00 ± 15.81	17.5
Type 2	2 (0.9%)	45.00 ± 28.28	45
Type 3	53 (23.6%)	13.68 ± 17.55	10
Type 4	25 (11.1%)	17.00 ± 19.04	5
Type 5	59 (26.2%)	18.39 ± 17.92	15
Type 6	5 (2.2%)	14.00 ± 10.84	15
Type 7	77 (34.2%)	19.68 ± 19.91	15
Total	225 (100%)	17.73 ± 18.69	10

Linear regression analysis did not reveal a statistically significant association between NSD types and the total NOSE score (Table 4).

**Table 4:** Results of linear regression analysis.

Parameter	B (95% Confidence Interval for B)	*p*-value
Mladina's types of NSD	0.837 (−0.628 to 2.301)	0.261
Angle of NSD	−0.122 (−0.859 to 0.615)	0.745

### Association between NSD angle and NOSE scores

The estimated angle of NSD ranged from 2.5° to 22.6° with a mean value of 8.6° ± 3.4 (mean ± SD). Linear regression analysis did not find a statistically significant influence of the NSD angle on the subjective perception of nasal obstruction (Table 4). It was interesting that the mean NSD angle was slightly higher in the group of patients who did not complain of nasal obstruction (Table 5).

**Table 5:** The angle of NSD in patients with and without nasal obstruction.

Nasal obstruction	Number of patients (%)	The angle of the NSD		
		Mean ± SD	Median	Min−Max
Absent (total NOSE score = 0)	58 (25.8%)	9.08° ± 3.81	8.8°	2.9°−22.6°
Present (total NOSE score > 0)	167 (74.2%)	8.5° ± 3.24	8.1°	2.5°−22.4°

## Discussion

The prevalence of NSD in the adult population varies among different studies. In our sample, the prevalence of the NSD was slightly higher in comparison to previous studies. Similar inter-study differences in the NSD prevalence also exist in relation to gender and Mladina's types of NSD. A few authors have reported a slightly higher NSD prevalence in males.^1^, ^16^, ^17^ In our study, however, NSD was more commonly encountered in females (60.4%), although this gender difference was not statistically significant. This could be due to the higher number of females in the study sample because participants were consecutively enrolled as they came to the CT examination. Our results also showed a predominance of Mladina's type 7, type 5, and type 3 NSD in decreasing order (Table 2). By contrast, an international study conducted by Mladina et al.^1^ found type 3 as the most prevalent (20.4%), followed by type 2 (16.4%) and type 1 (16.2%). Type 7 in their sample was the least frequent.^1^ However, type 1 NSD has been the most frequently diagnosed in Koreans, Indians, and Saudi Arabs.^16^, ^17^, ^18^

Potential reasons for the significant discrepancy in NSD frequencies between studies might be due to different techniques used to diagnose NSD, and due to inter-study differences in target populations. Techniques such as anterior rhinoscopy and endoscopy were frequently-used diagnostic tools for NSD. The disadvantage of anterior rhinoscopy is that posterior parts of the nasal septum cannot be fully visualized, and consequently, some posterior deviations might be omitted. Therefore, it could be expected that studies in which anterior rhinoscopy was an examination technique of choice underestimate real prevalence of NSD. Although endoscopy allows visualization of the complete nasal septum, the angle of the NSD could not be measured with great accuracy and repeatability.^9^, ^19^ Concerning the types of NSD, Mladina pointed out that type 5 can be easily overlooked by anterior rhinoscopy.^1^ A higher percentage of type 5 NSD in our sample could be explained by a more comprehensive evaluation of the nasal septum by CT.

Although CT is not recommended for routine NSD diagnosis and evaluation, it is superior to anterior rhinoscopy and endoscopy because it visualizes the whole septum in three planes. Thus, septal morphology can be carefully examined, the angle of NSD can be precisely measured, and none of the seven types of NSD can be misjudged. It also allows an accurate measurement of the nasal valve angle, which is essential for the differentiation between type 1 and 2 NSD. The CT assessment of the nasal valve angle could be the reason for a different frequency of type 1 and type 2 in the current study in comparison to other studies.

Previous studies that investigated NSD prevalence were conducted in ENT clinics where the number of patients that suffer from NSD can be significantly higher. Furthermore, it might falsely present harsh nasal obstruction symptoms in the majority of patients with NSD. However, it is well known that not all patients with NSD have nasal obstruction symptoms.^2^, ^8^ Out of 225 patients in the current study, symptomatic NSD was recorded in 167 (74.2%) patients. Therefore, we focused on a normal unbiased population, who presented with a wide range of NOSE scores and CT morphology of the nasal septum. Patients were selected at the Department of Diagnostic Radiology in order to target the most general population. This way, a more representative sample was obtained by omitting ENT clinics and implementing well-defined exclusion criteria. Therefore, it was possible to accurately investigate the real presence and severity of NSD induced nasal obstruction and whether different morphological characteristics of NSD influence the nasal obstruction symptoms.

According to NOSE scores, there were measurable differences in nasal obstruction severity between seven types of NSD. Patients with type 2 NSD reported the worst NOSE scores. Since this result was obtained from only two patients, it could not be simply extrapolated to the general population. However, these were not the only cases with type 2 NSD. Almost one-third of the type 7 NSD cases consisted of type 2 and other types of NSD (23/77 patients). Their NOSE scores were also relatively high, but the mean NOSE score for type 7 NSD was lower. Our finding was in accordance with previous clinical studies as well as the airflow dynamics analysis of the nasal valve region.^17^, ^20^, ^21^ Many authors argue that deviation in the nasal valve area is critical for nasal obstruction and causes the most burdensome obstruction symptoms. The mechanism behind this was revealed in studies that evaluated nasal airflow resistance in experimental nasal models. Namely, it has been found that constriction in the valvular region results in a higher increase in airflow resistance than narrowing in the middle of the nasal cavity.^21^ If there is a combined narrowing in the nasal valve region and the middle nasal cavity (type 7 NSD in Mladina's classification system), the valvular deviation usually has a more significant impact on nasal obstruction.^21^ Similar to type 2, the nasal valve angle is also changed in type 1. Patients with this NSD type also reported more severe symptoms than other NSD types (Table 3). However, observed differences in NOSE scores between Mladina's types of the NSD were not statistically confirmed as significant. Such a result could be a consequence of a relatively low number of patients with type 1 and 2 NSD compared to the percentage of other NSD types. This finding may reflect a true low prevalence of isolated types 1 and 2 in our population and/or their coexistence with other types that constitute type 7 NSD.

Based on the NOSE scores, it seems that type 5 is also important for the severity of the obstruction in our patients (Table 3). The presence of a bony spur characterizes this type of NSD. Wee et al.^17^ reported that, after type 1 and 2, patients with type 5 NSD frequently complained of nasal obstruction, but the symptom severity was not quantified. In general, the impact of spurs on the nasal obstruction was under investigated. Simmen et al.^22^ observed turbulent flow in an experimental cadaveric model of the nose behind the spur. In the computational model of the nasal cavity, Liu et al.^20^ found complicated airflow and velocity distribution associated with a spur, but the results were not presented in the paper.

The association between the angle of the NSD and nasal obstruction is not clear. Ardeshirpour et al.^10^ found out that the angle of deviation measured at the anterior, middle, and posterior part of the nasal septum, as well as maximal angle, poorly correlate with NOSE scores. The same author also noticed a poor connection between the more obstructed side of the nose and the side of the deviation.^10^ The opposite conclusion was provided by Lee and his team^9^ who stated that the angle of NSD measured at the osteomeatal unit level has a significant impact on the subjective symptoms of nasal obstruction. However, the same author failed to detect any connection between nasal valve angle and obstruction symptoms.^9^ According to Savovic et al.,^8^ a NSD angle greater than 10° has a significant effect on the difficulty of breathing through the deviated side of the nose. In our study, the NSD angle ranged from 2.5° to 22.6°, but regression analysis did not show its significant effect on nasal obstruction. The fact that a slightly higher mean and maximum NSD angle was measured in patients without nasal obstruction symptoms supports this conclusion (Table 5).

## Conclusions

The estimated prevalence of NSD in the Serbian population was high. The most common type of NSD was type 7, while the least frequent was type 2. Patients in the current study showed apparent differences in NOSE scores in relation to the type of NSD. Our results suggest that morphological characteristics of NSD expressed through the Mladina's classification and the NSD angle could not fully explain nasal obstruction severity.

## Conflicts of interest

The authors declare no conflicts of interest.
